# Risk for surgical positioning injuries: scale validation in a rehabilitation hospital*

**DOI:** 10.1590/1518-8345.2912.3261

**Published:** 2020-05-11

**Authors:** Francisca Caroline Lopes do Nascimento, Maria Cristina Soares Rodrigues

**Affiliations:** 1 Rede Sarah de Hospitais de Reabilitação, Centro Cirúrgico, Brasília, DF, Brazil.; 2 Universidade de Brasília, Departamento de Enfermagem, Brasília, DF, Brazil.

**Keywords:** Perioperative Nursing, Patient Safety, Patient Positioning, Risk Assessment, Nursing Care, Wounds and Injuries, Enfermagem Perioperatória, Segurança do Paciente, Posicionamento do Paciente, Medição de Risco, Cuidados de Enfermagem, Ferimentos e Lesões, Enfermería Perioperatoria, Seguridad del Paciente, Posicionamiento del Paciente, Medición de Riesgo, Atención de Enfermería, Heridas y Lesiones

## Abstract

**Objective:**

to validate the Risk Assessment Scale for the Development of Injuries due to Surgical Positioning in the stratification of risk for injury development in perioperative patients at a rehabilitation hospital.

**Method:**

analytical, longitudinal and quantitative study. An instrument and the scale were used in the three perioperative phases in 106 patients. The data were analyzed using descriptive and inferential statistics.

**Results:**

most patients showed high risk for perioperative injuries, both in the scale score with estimated time and in the real-time score, with a mean of 19.97 (±3.02) and 19.96 (±3.12), respectively. Most participants did not show skin lesions (87.8%) or pain (92.5%). Inferential analysis enabled us to assert that the scale scores are associated with the appearance of injuries resulting from positioning, therefore, it can adequately predict that low-risk patients are unlikely to have injuries and those at high risk are more likely to develop injuries.

**Conclusion:**

the scale validation is shown by the association of scores with the appearance of injuries, therefore, it is a valid and useful tool, and it can guide the clinical practice of perioperative nurses in rehabilitation hospitals in order to reduce risk for injuries due to surgical positioning.

## Abstract (pt)

**Objetivo:**

validar a Escala de Avaliação de Risco para o Desenvolvimento de Lesões Decorrentes do Posicionamento Cirúrgico na estratificação do risco de desenvolvimento de lesões em pacientes perioperatórios de um hospital de reabilitação.

**Método:**

pesquisa analítica, longitudinal e quantitativa. Um instrumento e a escala foram utilizados nas três fases perioperatórias em 106 pacientes. Os dados foram analisados por estatística descritiva e inferencial.

**Resultados:**

a maioria dos pacientes apresentou alto risco para lesões perioperatórias, tanto no escore da escala com tempo estimado quanto no escore com tempo real, com média 19,97 (+3,02) e 19,96 (+3,12), respectivamente. A maioria dos participantes não apresentou lesão de pele (87,8%) e dor (92,5%). A análise inferencial permite afirmar que os escores da escala estão associados com o surgimento de lesões decorrentes do posicionamento, portanto, consegue prever adequadamente que pacientes com baixo risco provavelmente não apresentarão lesões e aqueles com alto risco estarão mais propensos a desenvolver lesões.

**Conclusão:**

a validação da escala é evidenciada pela associação dos escores com o surgimento de lesões, portanto, é ferramenta válida e útil, e poderá nortear a prática clínica do enfermeiro perioperatório de hospital de reabilitação na redução de riscos de lesões decorrentes da posição cirúrgica.

## Abstract (es)

**Objetivo:**

validar la Escala de Evaluación de Riesgo para el Desarrollo de Lesiones Debidas al Posicionamiento Quirúrgico en la estratificación del riesgo de desarrollo de lesiones en pacientes perioperatorios de un hospital de rehabilitación.

**Método:**

investigación analítica, longitudinal y cuantitativa. Se utilizó la escala y un instrumento en las tres etapas perioperatorias en 106 pacientes. El análisis de datos se realizó con estadística descriptiva e inferencial.

**Resultados:**

la mayor parte de los pacientes presentó alto riesgo de desarrollo de lesiones perioperatorias, tanto en el puntaje de la escala con tiempo estimado como en el puntaje de tiempo real, con promedio 19,97 (+3,02) y 19,96 (+3,12), respectivamente. La mayor parte de los participantes no presentaron lesiones de piel (87,8%) ni dolor (92,5%). El análisis inferencial permite afirmar que los puntajes de la escala están asociados con el surgimiento de lesiones provenientes del posicionamiento, por lo tanto, se infiere que los pacientes con bajo riesgo probablemente no presenten lesiones y que, aquellos de alto riesgo, serán más proclives a desarrollar lesiones.

**Conclusión:**

la validación de la escala se evidencia por la asociación de los puntajes con el surgimiento de lesiones, por tanto, es una herramienta válida y útil, y podrá guiar la práctica clínica del enfermero perioperatorio del hospital de rehabilitación en lo atinente a la reducción de riesgos de lesiones provenientes de la posición quirúrgica.

## Introduction

Surgical positioning is a key factor in the performance of safe and efficient operative procedures, and it aims to provide the best anatomical exposure for surgery, although there are risks to patients that result from the position adopted on the operating table. All positions present risks that may be exacerbated, as the patient is under anesthesia and, in most cases, unable to alert the team about his or her discomfort^(^ 1 ^-^ 2 ^)^.

Fixed positioning associated with prolonged surgery time can cause bone pressure points against the operating table and cause temporary or even permanent damage to the patient^(^ 2 ^-^ 3 ^)^.

Pressure injuries (PIs) due to surgical positioning are considered complications, and they have a multifactorial etiology. Despite technological advances, they are still a challenge for clinical practice. The adoption of adequate protective measures is compromised by the difficulty that the surgery team has in the early assessment of risk in surgical patients^(^ 4 ^)^.

With the premise of promoting safe and quality care, comfort and individuality for each patient, perioperative nurses are responsible for planning nursing actions that can reduce and prevent complications resulting from the anesthetic-surgical procedure, thus minimizing potential risks. Therefore, they must provide adequate surgical positioning, with the equipment and devices that are available and appropriate to help the performance of the procedure and, thus, implement effective interventions^(^ 2 ^-^ 3 ^)^.

In order for interventions to be effective in preventing skin injuries, they must be related to pressure relief while and immediately after the patient remains on the operating table, and examples of effective devices for such prevention are dry viscoelastic polymer mattress toppers and gel pads^(^ 5 ^)^.

In the national literature, studies show the occurrence of PI related to surgical positioning, such as one involving 199 surgical patients with the presence of PI in 20.6% of the sample. In most cases (98.6%), the injuries were in stages 1 and 2^(^ 6 ^)^. Another study, conducted in a university hospital, showed the occurrence of 25% PI in a total of 148 patients who underwent elective surgery^(^ 7 ^)^.

Even more worrying results are shown in a study conducted with 50 patients evaluated when admitted to the operating room (OR) and immediately after the surgical intervention, identifying that 37 patients (74%) had stage-1 injuries and that on only one patient were protection resources used^(^ 8 ^)^. Another study identified that out of 115 patients who underwent elective surgery, 46 (40%) had pain due to surgical positioning, and 25 (21.7%) developed PI^(^ 2 ^)^.

Injuries related to peripheral nerves or peripheral neuropathies are an uncommon complication of surgery, with estimates ranging from 0.02% to 21%^(^ 9 ^)^. A systematic review of 23 studies that evaluated sensory changes or nerve damage after an abdominoplasty reported that most injuries occurred when surgery included more than one type of procedure and also suggested that patient risk increased with surgery time^(^ 10 ^)^.

Early risk assessment, including the use of a combination of a validated risk assessment instrument, skin assessment and clinical judgment, is crucial^(^ 11 ^)^. Recently, a study showed the importance of establishing a specific risk scale for surgical patients, since the same study compared existing scales which assess PI development and showed that they were not so effective as they did not identify the critical factors of the perioperative period^(^ 2 ^)^.

The Risk Assessment Scale for the Development of Injuries due to Surgical Positioning (ELPO), developed by a Brazilian researcher, includes domains and items that, according to the literature, represent greater or lesser risk for the development of injuries due to patients’ surgical positioning. ELPO was based on the evidence available in the literature and organized by the anatomical and physiological implications of surgical positions on the patient’s body^(^ 2 ^)^.

However, an instrument that is designed and validated for a given environment does not always become applicable in different organizations. Thus, this study was performed aiming at the improvement of a protocol for the prevention of injuries due to the surgical positioning of patients at a rehabilitation hospital by considering the profile and specificity of the patients assisted and the hospital characteristics.

The objective of the study was to validate the Risk Assessment Scale for the Development of Injuries due to Surgical Positioning in the stratification of risk for injury development in perioperative patients at a rehabilitation hospital.

As a result of achieving this purpose for the clinical practice of perioperative nurses, the possibility of obtaining a valid and useful ELPO was attempted so that it could be applied in the context of care provision for surgical patients in a rehabilitation hospital as a tool available for efficient management in decision making regarding injury prevention, in addition to contributing to improvement in this field of nursing know-how through the scientific support obtained from the study of the problem in question.

## Method

This is an analytical, longitudinal quantitative study. Data were collected in large quaternary rehabilitation hospital which is a national reference in rehabilitation and located in the city of Brasília, Federal District (FD), Brazil.

The hospital’s surgery facilities (SF) have eight operating rooms, each consisting of an anesthetic induction room, where anesthetic procedures are performed and the patient is prepared for the surgical operation. According to recommended practices, the SF should have an anesthetic induction area in its composition, however, this does not hold true for most Brazilian hospitals. The international literature points out that this area, in addition to being used for anesthetic procedures and patient preparation, is a positive factor for the patient, as it promotes a calm environment for the start of procedures^(^ 12 ^)^.

The study was carried out at the SF and in the wards that receive inpatients in the pre- and postoperative periods from January to February 2018. The permanent staff on the service’s nursing team, which provides direct care for surgical patients, comprised 24 nurses, 13 nursing technicians and three nursing assistants, totaling 40 employees. Among nurses, one was responsible for receiving each patient at the SF and monitoring anesthetic procedures; therefore, he/she was directly responsible, together with the other team members, for the patient’s positioning and ELPO application, thus being a differential in the perioperative care provided.

To calculate the representative sample size, the GPower 3 software was used with the following parameters: two-tailed correlation test, 80% test power, 5% error probability and average effect size. Thus, the number of 82 participants was obtained to seek the internal validity of the study. It was a convenience sample, and 106 patients participated in the study, that is, a larger sample than the estimated minimum was achieved.

The target population in the study consisted of surgical patients undergoing elective procedures, of both sexes, aged 18 years or over, from any surgical specialty. Patients undergoing a second surgical procedure within the data collection period and patients undergoing emergency procedures were excluded.

The data source was primary, and a data collection instrument (the same as that used by ELPO’s author)^(^ 2 ^)^ as well as ELPO were applied. In the instrument, pre- and postoperative patient information regarding the patient’s characterization, skin integrity and presence of pain was recorded. A change to the original instrument was required, since the field where the grade assigned to the patient regarding the Braden scale was modified to include the ELPO grade, as this study does not aim to compare the predictive value of the aforementioned scales. The use of the instrument and the modification performed were authorized by the its author.

ELPO contains seven items (type of surgical position, length of surgery, type of anesthesia, support surface, limb position, comorbidities and patient’s age) with five sub-items each. The score ranges from one to five points and the total score from seven to 35 points. Patients with up to 19 points are considered to be at low risk, and those with 20 points or more are at high risk. The higher the score according to which a patient is classified, the greater the risk for developing injuries due to surgical positioning^(^ 2 ^)^.

Prior to data collection, a nurse from SF, who was previously trained, was invited to assist during the preoperative visit stage. Then, a pre-test involving 10 patients (not included in the sample) was carried out to evaluate the applicability of the proposed instruments as well as the adequacy of the dynamics to be adopted in the development of the study.

The research took place in the perioperative phases: 1) Preoperative period: nursing visit to the infirmary, when the instrument was used to record sociodemographic data and intrinsic factors to the patient as well as to take notes on aspects related to skin inspection and the assessment of the presence of pain; 2) Intraoperative period: ELPO application by the nurse in the operating room (OR); 3) Postoperative period: evaluation of the appearance of possible injuries (outcome), represented, in this study, by skin injury (reactive hyperemia and pressure injury) and the presence of pain related to surgical positioning. The presence of pain was assessed preoperatively for final comparison, in the postoperative period, if it did not exist and was related to surgical positioning.

The Numerical Rating Scale (NRS) was used to measure pain intensity, by which pain intensity is quantified using numbers, from 0 to 10, where point 0 represents no pain and 10 represents the worst possible pain. It can be applied graphically or verbally, and the respondent chooses the number that best represents his or her pain^(^ 13 ^)^.

For data collection in the preoperative period, the selection of potential patients participating in the study was carried out the day before hospital admission, according to the established inclusion criteria, after the surgical map was assessed. The researcher and the auxiliary nurse carried out the preoperative visit and, after filling in the pertinent data for patients’ characterization, they performed their skin inspection, recorded the presence of pain (type, location and intensity) through NRS and assessed the existence of physical limitation.

In the intraoperative period, ELPO was applied, and its score was recorded by the anesthetic induction nurse with assistance from the researcher in charge, who observed the surgical position of each patient. When the patient’s positioning was concluded, the nursing team member circulating in the operating room (OR) recorded the presence or absence of injury, which was evaluated by the researcher in the post-anesthesia recovery room (PARR).

At the end of each day, each patient’s score was checked, and the points generated by the items on the scale were evaluated so that there were no differences of opinion. ELPO was applied with the estimated surgical time, which was considered in this study as ELPO 1. It was, then, applied again with the real positioning time and designated as ELPO 2. This procedure enabled the comparison of the means obtained in each score, since one of the most significant risk factors is the time that patients remain on the operating table, as they may be subjected to intense and prolonged pressure during long surgical procedures, which creates a risk for developing PI^(^ 2 ^,^ 14 ^)^.

In the postoperative period, the researcher performed the skin inspection and recorded the evaluation on the instrument in the immediate postoperative period (IPO) and up to the limit of four postoperative days (PO) or until the injury appearance (outcome), if that happened first.

The data were analyzed using the IBM Statistical Package for Social Sciences (SPSS), version 20.0. The sample was characterized through descriptive analysis with absolute and percent frequency, mean and standard deviation. Comparisons of the means found in ELPO 1 and ELPO 2 were performed using the paired Student’s t-test. To evaluate the association of ELPO scores with the appearance of injuries due to positioning, the Chi-square test of independence was applied with Monte Carlo simulation and *Post-hoc* analysis with the Bonferroni method, when necessary. The level of significance adopted was p-value <0.05.

The research project was approved by the Research Ethics Committee of the hospital where the study was carried out, with CAAE no. 72695317.4.0000.0022 and approval report no. 2.343.997/2017, in compliance with Resolution 466/2012 by the National Health Council. All participants signed the Informed Consent Form.

## Results

One hundred and six patients participated in the study, of whom 54 (50.9%) were females, with a mean age of 46.36 years (±16.32) and a mean Body Mass Index (BMI) of 27.79 (±4.81). Most participants were employed (n=73; 68.9%) and came from the Federal District (n=61; 57.5%).

In the preoperative period, 88 patients (83.0%) did not show any pain complaints that were not related to the surgical site, 105 (99.1%) had intact skin, 99 (93.4%) had no history of PI, and 57 (53.8%) showed no physical limitations. With regard to comorbidities, most patients had more than one comorbidity, however, the comorbidity with the highest score in ELPO was considered, according to the instructions for the scale use. It was found that 61 (57.5%) of the patients had a neuropathy, 24 (22.6%) showed no comorbidities, 15 (14.2%) were obese, four (3.8%) had vascular disease, and two (1.9%) were diabetic.

As for surgical specialties, there was a higher frequency of orthopedic procedures, with 51 surgeries (48.1%), followed by neurosurgery, with 39 surgeries (36.8%), 12 (11.3%) plastic surgeries, three (2.8%) urological surgeries and one (1%) thoracic surgery.

The patients’ intraoperative data regarding the type of surgical position, limb position, duration of surgery, type of anesthesia and type of support surface are shown in Table 1.

**Table 1:** Distribution of rehabilitation patients according to the type of surgical position and the position of the limbs. Brasília, FD, Brazil, 2018

Position	n	%
Surgical Position		
Supine	67	63.2
Prone	26	24.5
Lateral	12	11.3
Lithotomy	1	1.0
Limb Position		
Opening of UL* < 90º	65	61.3
Knees raised < 90º and opening of LL^†^ < 90º or neck without chin-esternal alignment	27	25.5
Anatomic position	14	13.2
Total	106	100.0

* UL = Upper Limbs;

† LL = Lower Limbs

Regarding the type of surgical position, the supine position was the most frequent in surgical procedures (n=67; 63.2%), and regarding the position of the limbs, 65 (61.3%) were positioned with their upper limbs open at an angle that was less than 90º.

As for surgery duration, the frequencies were distributed as follows: seven patients (6.6%) underwent surgery for up to 1 hour; 33 (31.1%) for over 1h to 2h; 37 patients (34.9%) for over 2h to 4h; 20 patients (18.9%) underwent procedures from 4 a.m. to 6 a.m.; and nine (8.5%) remained in surgery for over 6h.

In the investigated sample (n=106), 49 (46.2%) underwent general anesthesia, 33 (31.1%) received general + regional anesthesia, 22 (20.8%) received only regional anesthesia, and two (1.9%) had sedation as anesthesia.

During the pre-test, it was necessary to group the available resources and equipment and the way they are distributed to assemble the Support Surfaces (SS) for each type of patient so that the nursing team could understand it. Thus, they were distributed within each item proposed by the scale (Table 2).

**Table 2:** Distribution of SS* used for the positioning of surgical patients in the rehabilitation hospital. Brasília, FD, Brazil, 2018

SS*	n
No use of SS* or rigid supports without padding or narrow leg holders	
Rigid-surface operating table	15
Foam operating table mattress (conventional) + cotton-field cushions	
Cotton-field cushions + aspirated memory-foam mattress	6
Padded cotton-field cushions	3
Cotton-field cushions + viscoelastic mattress	2
Foam operating table mattress (conventional) + foam cushions	
Conventional foam operating table mattress	35
Aspirated memory-foam mattress + pillow	12
Non-aspirated memory-foam mattress + pillow	8
Mayfield + pillow	7
Foam operating table mattress (conventional) + pillow	1
Foam operating table mattress (conventional) + viscoelastic cushions	
Viscoelastic gel cushion + pillow	7
Viscoelastic operating table mattress + viscoelastic cushion	
Viscoelastic operating table mattress + pillow	7
Viscoelastic surgical saddle + viscoelastic gel cushion	3

* SS = Support Surface

Regarding the type of SS used to position the patient, of the 106 procedures analyzed, 63 (59.4%) used a foam operating table mattress (conventional) + foam cushions (Table 3).

**Table 3:** Distribution of rehabilitation patients, according to the type of SS*. Brasília, FD, Brazil, 2018

SS*	n	%
Foam operating table mattress (conventional) + foam cushions	63	59.4
No use of SS* or rigid supports without padding or narrow leg holders	15	14.2
Foam operating table mattress (conventional) + cotton-field cushions	11	10.4
Viscoelastic operating table mattress + viscoelastic cushion	10	9.4
Foam operating table mattress (conventional) + viscoelastic cushions	7	6.6
Total	106	100.0

* SS = Support Surface

In the study sample, 10 (9.4%) patients showed reactive hyperemia in the areas of the forehead, chin, interscapular region, anterior chest, iliac crest, trochanteric region and knees; three (2.8%) had stage-1 PI in the chin region and left side of the forehead, sacral region, chin and right side of the chest; and 93 (87.8%) had no injuries.

As for pain related to surgical positioning, eight (7.5%) complained of pain located in the shoulders (n=3), arm (n=2), right side of the chin and right side of the chest (n=1), neck (n=1) and sacral region (n=1). In evaluating this variable, 92.5% (n=98), that is, the majority, did not report any pain due to surgical positioning.

When the ELPO score was applied, in ELPO 1, there was a frequency of 48 patients (45.3%) at low risk of developing injury and 58 (54.7%) at high risk, and in ELPO 2, there was a frequency of 49 patients (46.2%) at low risk and 57 (53.8%) at high risk. In both ELPO 1 and ELPO 2, there was a predominance of patients at high risk for developing injuries, with a mean of 19.97 (±3.02) and 19.96 (±3.12), respectively.

The comparison of the means of ELPO and ELPO 2 scores showed an inferential analysis that enables us to state that there is no difference between the scores obtained at the two moments: *t* (105) = 0.120; p = 0.905.

Table 4 shows the association of ELPO 1 and ELPO 2 scores with the appearance of injuries due to surgical positioning (outcome). Inferential analysis allows us to state that both the ELPO 1 score (χ^2^ (1) = 12.268; p < 0.001; n = 106) and that of ELPO 2 (χ^2^ (1) = 8.851; p = 0.002; n = 106) scores are associated with the appearance of a skin lesion, and it also points out that the scores of ELPO 1 (χ^2^ (1) = 7.161; p = 0.006; n = 106) and ELPO 2 (χ^2^ (1) = 3.960; p = 0.048; n = 106) are significantly associated with the presence of pain resulting from surgical positioning.

**Table 4:** Results of the Chi-square test for the association of ELPO scores* with the appearance of injuries resulting from the surgical positioning of rehabilitation patients. Brasília, FD, Brazil, 2018

ELPO*	n 106	Skin injury				Pain		
		Yes n=13	No n=93	p-value^†^		Yes n=8	No n=98	p-value^†^
ELPO* 1								
Low Risk	48	0	48	<0.001^‡^		0	48	0.006^‡^
High Risk	58	13	45			8	50	
ELPO* 2								
Low Risk	49	1	48	0.002^‡^		1	48	0.048^‡^
High Risk	57	12	45			7	50	

* ELPO = Risk Assessment Scale for the Development of Injuries due to Surgical Positioning;

† p-value;

‡ significance test (p-value) referring to the calculation of the Chi-Square of independence; significance level: p<0.05.

## Discussion

When evaluating patients undergoing elective surgery, it was found that the mean age was 46.36 years (±16.32) and that the mean BMI was 27.79 (±4.81). The literature shows that the incidence of complications increases proportionally to age, with less tolerance to prolonged positioning. It also increases proportionally to obesity because, depending on the type of position, it favors the compression of the diaphragm and hinders chest expansion^(^ 15 ^-^ 16 ^)^. Changes in BMI (underweight, overweight or obesity) influence the appearance of injuries caused by surgical positioning^(^ 1 ^)^.

Another aspect found was that the majority of patients did not report pain, had intact skin, with no history of PI or physical limitations. Physical limitation was established so that, at the time of positioning, there would be available resources and the surgical position would be in accordance with the patient’s tolerance.

As for the presence of comorbidities, a factor in which most patients had two or more associated diseases, it is noteworthy that some diseases imply the fragility of the patient’s body systems, such as vascular and respiratory diseases, neuropathies and even malnutrition, and the more severe they are, the greater the risk for developing injuries^(^ 8 ^)^.

Diabetes *mellitus* causes impaired tissue perfusion to the patient due to decreased blood flow, which makes healing difficult and is considered a risk factor for the occurrence of perioperative lesions due to positioning^(^ 17 ^)^. Corresponding to the characteristic of the hospital where the study was conducted, the surgical specialty of orthopedics showed a higher frequency of surgeries.

Some positions were analyzed during the intraoperative period, the most frequent being the supine position, followed by the prone position. The patients predominantly remained with the upper limbs open at an angle less than 90º. The supine position, in this study, is the position chosen for anesthetic induction, and the patient remains in the same position until the end of the surgical procedure. It is the position that mostly respects body alignment, and complications only occur in cases where positioning is improperly performed and/or when the patient remains in such position for a long time as a result of the pressure points against the operating table^(^ 18 ^)^.

When the patient is in the supine position, with his or her arms in bracers, they must be supinated (with the palms facing upwards), the bracers must be levelled with the mattress and the arms must be abducted at an angle less than 90º in order to avoid possible discomfort and improper positioning^(^ 3 ^)^. The prone position can cause complications that are considered potentially serious due to vascular compression, hemodynamic changes, increased abdominal pressure and PI^(^ 19 ^)^.

Long periods of immobilization and pressure exposure cause anoxia, tissue necrosis and consequent skin damage; therefore, the duration of the anesthetic-surgical procedure in the intraoperative period is characterized as one of the most significant risk factors and as a contributor to the appearance of injuries due to surgical positioning^(^ 3 ^)^. The longer the surgery, the greater the chance of developing PI, and the prevalence rate of PI in patients who undergo surgery lasting more than three hours is 8.5% or more^(^ 20 ^)^. The risk for the patient’s developing this type of injury increases by 1.07 every hour of surgery^(^ 21 ^)^.

The type of anesthesia is another significant risk factor in the intraoperative period, since it depresses pain receptors, influences the level of depression of the nervous system and relaxes the muscles, causing the patient’s defense mechanisms to no longer provide protection against pressure, stretching, muscular effort and/or damage resulting from the exacerbated rotation of the limb, making it susceptible to pressure injury and pain^(^ 5 ^)^.

In order to provide adequate and safe positioning to the patient, it is necessary to use supports and cushions as well as decrease height during leg elevation; however, the availability and appropriate selection of support surfaces (SS) are mainly required^(^ 5 ^)^.

SS are specialized devices used in order to redistribute pressure. They are designed for the management of tissue pressure by reducing the shear force and controlling the local microclimate. Thus, they must be chosen according to patients’ specific needs and surgery type^(^ 22 ^)^.

Failure to use SS during the intraoperative period increases the risk for injuries resulting from surgical positioning, as found in a systematic review^(^ 22 ^)^. However, SS are not regularly used on surgical patients due to political, economic and social issues faced in the country, thus, in many public services, such SS are not available, which interferes in the prevention of these injuries^(^ 5 ^)^.

In the study, the SS used for the patients were analyzed and, for most of them, a foam operating table mattress (conventional) + foam cushions were used due to the type of position chosen for the procedure. The hospital has adequate positioning resources, and it is up to the nurse to choose the SS that mostly reduce, relieve and redistribute pressure, considering the specific needs of each patient and the chosen position.

The national literature shows evidence of a relatively high incidence of injuries resulting from surgical positioning, mainly PI. In this study, reactive hyperemia, a type hyperemia that is blanchable to finger pressure and that usually disappears in less than an hour, was considered, since the lack of pressure relief results in tissue ischemia or anoxia, thus causing PI^(^ 15 ^)^.

Therefore, the incidence of injuries resulting from surgical positioning, compared to that in other studies, was considered low and a result of the quality of the care provided, since the hospital works with a larger number of nurses who are directly responsible for surgery patients. Additionally, the SF follow recommended norms and standards and have resources and equipment available to provide adequate and safe positioning.

The use of an evaluation scale that includes intrinsic and extrinsic risk factors in surgical-trauma and surgical-injury surgery can help nurses to identify patients at higher risk at an early stage. The use of ELPO is an important step in preventing complications. With the use of this type of tool, nurses can plan the implementation of effective solutions in the intraoperative period so that patients are not affected by such injuries^(^ 2 ^)^.

This study showed that in both ELPO 1 (54.7%) and ELPO 2 (53.8%), patients were at high risk for developing injuries due to surgical positioning. It is noteworthy that for each additional point on the scale with which a patient is classified, the probability of developing an injury increases by 44%^(^ 23 ^)^.

According to recommendations for ELPO use and application, as regards the item related to surgery time, such time should be estimated. When comparing the averages of the scores obtained by ELPO 1 and ELPO 2, there is no significant difference between the scores obtained at both times, that is, the scores of ELPO 1 and ELPO 2 are equivalent, hence it is inferred that the scale can be applied using the estimated time.

In order to evaluate the association of ELPO scores with the appearance of injuries related to patients’ surgical positioning, the injuries investigated were the development of PI and the presence of pain. The results indicated that both ELPO 1 scores and ELPO 2 scores are associated with the appearance of skin lesions and the presence of pain. Therefore, ELPO is able to adequately predict that individuals who are at low risk are unlikely to have skin injury and pain, and those who are at high risk are, in fact, more likely to develop skin injury and pain due to surgical positioning.

This study has some limitations. The investigation was conducted in a quaternary-level hospital which serves patients with specific characteristics, that is, patients in rehabilitation or who may develop some type of physical limitation. It has a differentiated nursing team that comprises a larger number of nurses in relation to other Brazilian hospitals and, in addition, it has adequate and appropriate positioning resources to provide quality care and patient safety. Therefore, this study can be replicated in services with similar characteristics, and the results obtained can contribute with evidence that will collaborate to the development of this field of knowledge and care provision.

With the advancement of technology, new surgical techniques are being assimilated, resulting in a consequent adaptation in patient positioning. Therefore, there is a need to acquire new positioning resources, especially pressure relief devices. With this regard, the items proposed by the scale require a review for the adequacy and viability of the instrument so that it can adapted to different care-provision realities.

## Conclusion

The ELPO applied in this study proved to be a valid and useful instrument for assessing the risk of developing injuries resulting from surgical positioning in adult perioperative patients at a rehabilitation hospital, as shown by the association of ELPO 1 and ELPO 2 with the appearance of injuries due to surgical positioning.

In clinical practice, the use of ELPO in rehabilitation hospitals will promote the improvement of perioperative care if it is included in a nursing care protocol aimed at the adequate and safe positioning of surgical patients.
